# Vortioxetine in the treatment of somatoform disorder: a case report

**DOI:** 10.1192/j.eurpsy.2026.11782

**Published:** 2026-07-31

**Authors:** T. Mastelić, E. Burilović, M. Ivandić, T. Vukićević, M. Milanović, M. Galetić Pavlov, T. Glavina, D. Lasić

**Affiliations:** Department of Psychiatry, University Hospital of Split, Split, Croatia

## Abstract

**Introduction:**

Vortioxetine is a newer multimodal antidepressant. It is usually used to treat depressive symptoms, and less often for anxiety symptoms. Somatoform disorder is characterized by persistent somatic symptoms of unclear etiology that cause distress and impair functioning. It is most commonly treated with selective serotonin reuptake inhibitors. Treatment is often challenging and only partially successful. Vortioxetine has so far shown success in the treatment of somatic symptoms of depression. According to available data, only one case report of two patients with somatoform disorder treated with vortioxetine has been published.

**Objectives:**

To report a patient with somatoform disorder who achieved complete remission with vortioxetine.

**Methods:**

Review of medical records.

**Results:**

A patient in his early 20s complains of longstanding fatigue that progresses. He describes apathy, lack of pleasure, and energy. He complains of a reduced intensity of emotions. The dominant concern is about his own condition. He describes physical and mental fatigue that can be so strong at times that he cannot get up. He reports walking with effort, needing to focus on each step. Mental effort quickly exhausts him, even when reading about interesting topics. Often, mental effort results in headaches. For all this, he had to suspend a year of studies at university. He describes a lack of sexual desire, pain after ejaculation, pain in the testicles, and the perineum. Urological, endocrinological, and neurological examinations did not reveal an organic substrate. The patient was titrated with vortioxetine from 5 mg to 20 mg over a month and a half. In the period of the next four months, he gradually achieved full recovery on a dose of 20 mg. At one point, duloxetine was added at a dose of 30 mg because recovery slowed, but due to increased irritability and later sleepiness, the patient spontaneously, on his own decision, stopped taking it, after which improvement returned. The patient is now three months in remission and continues to take the vortioxetine at a dose of 20 mg.

**Conclusions:**

We presented a case of a patient who fully recovered from somatoform disorder thanks to vortioxetine at a dose of 20 mg. Based on current knowledge, we postulate that this is due to the antagonistic action of vortioxetine on 5-HT3 and 5-HT1B/1D receptors, as well as inhibition of serotonin reuptake. There are also studies suggesting anti-inflammatory and analgesic effects of vortioxetine, which are also important in the treatment of somatoform disorder. Given the paucity of data on this topic, we believe that further research is needed and that this case report has its significance. 5-

**Disclosure of Interest:**

None Declared

